# Assessing the occurrence and status of wheat in late Neolithic central China: the importance of direct AMS radiocarbon dates from Xiazhai

**DOI:** 10.1007/s00334-019-00732-7

**Published:** 2019-06-03

**Authors:** Zhenhua Deng, Dorian Q. Fuller, Xiaolong Chu, Yanpeng Cao, Yuchao Jiang, Lizhi Wang, Houyuan Lu

**Affiliations:** 1 Center for the Study of Chinese Archaeology, Peking University, Beijing, 100871 China; 2 School of Archaeology and Museology, Peking University, Beijing, 100871 China; 3 Institute of Archaeology, University College London, 31-34 Gordon Square, London, WC1H 0PY UK; 4 School of Archaeology and Museology, Northwest University, Xi’an, 710069 Shaanxi China; 5 Henan Provincial Bureau of Cultural Heritage, Zhengzhou, 450002 Henan China; 6 Henan Provincial Institute of Cultural Relics and Archaeology, Zhengzhou, 450099 China; 7 School of History, Zhengzhou University, Zhengzhou, 450001 Henan China; 8 National Museum of China, Beijing, 100006 China; 9 Institute of Geology and Geophysics, Chinese Academy of Sciences, Beijing, 100029 China; 10 Center for Excellence in Tibetan Plateau Earth Science, Chinese Academy of Sciences, Beijing, 100101 China; 11 University of Chinese Academy of Sciences, Beijing, 100049 China

**Keywords:** Triticum aestivum, Bronze Age, Agriculture, Urbanisation, Silk road

## Abstract

The introduction of wheat into central China is thought to have been one of the significant contributions of interactions between China and Central Asia which began in the 3rd millennium bc. However, only a limited number of Neolithic wheat grains have been found in central China and even fewer have been directly radiocarbon dated, making the date when wheat was adopted in the region and its role in subsistence farming uncertain. Based on systematic archaeobotanical data and direct dating of wheat remains from the Xiazhai site in central China, as well as a critical review of all reported discoveries of Neolithic and Bronze Age wheat from this region, we conclude that many wheat finds are intrusive in Neolithic contexts. We argue that the role of wheat in the subsistence of the late Neolithic and early Bronze Age of central China was minimal, and that wheat only began to increase in its subsistence role in the later Bronze Age during the Zhou dynasty after ca. 1000 bc.

**Electronic supplementary material:**

The online version of this article (10.1007/s00334-019-00732-7) contains supplementary material, which is available to authorized users.

## Introduction

*Triticum* spp. (wheat), as an exotic crop, has been the most important dietary staple in northern China for thousands of years. At the present day its production, measured in terms of yield or land area, greatly exceeds the native millets *Setaria italica,* (foxtail millet) and *Panicum miliaceum* (broomcorn millet) (National Bureau of Statistics of China 2017). Wheat was domesticated in western Asia in the early Holocene (Zohary et al. 2012; Fuller et al. 2014; Fuller and Lucas 2014), and it was introduced into China after interactions with Central Asia that began in the middle of the 3rd millennium bc (Frachetti et al. 2010; Boivin et al. 2012; Liu and Jones 2014; Stevens et al. 2016; Spengler et al. 2016). Although several wheat species were domesticated in western Asia, including the diploid *Triticum monococcum* and tetraploids (notably *T. dicoccon* and *T. durum*), most of which spread through the Iranian and Indian regions and into southern Central Asia in prehistory, it was only *T. aestivum*, hexaploid, free-threshing bread wheat that is known traditionally or archaeologically in China (Fuller and Lucas 2014; Stevens et al. 2016). There remains continuing debate over the precise period of introduction of wheat to China, and considerable recent research effort into the timing and route (for example, Flad et al. 2010; Betts et al. 2014; Barton and An 2014; Zhou and Garvie-Lok 2015; Zhao 2015b; Liu et al. 2016; Stevens et al. 2016), as well as interest in why only this particular form of wheat was brought eastwards. In terms of timing, this comes down to a debate whether this took place before or after 2000 bc, one or more times, and by one or more routes. An additional area of debate is whether the early wheat in China was of little importance for subsistence and just a minor experimental crop (Boivin et al. 2012), or if wheat was already important in subsistence and diet (Liu and Jones 2014). Some of this debate, especially in terms of the importance of wheat, may derive from different agricultural patterns in various parts of China, calling for more regionally specific assessments of the evidence.

Generally, three different models have been proposed to explain the process of wheat introduction into China. The first hypothesis argues that wheat was brought from Central Asia through Xinjiang and then diffused from west to east within China, following the path of the traditional Silk Road (Li 2002). The second suggests a route by the steppes north of the Tian Shan mountains and into the Hexi corridor via the Heihe river (Black river), and from there westwards into Xinjiang and eastwards into the middle and lower Huang He (Yellow river) valley (Flad et al. 2010; Dodson et al. 2013). A variant of this hypothesis offers to explain the nearly synchronous appearance of wheat in western, central and eastern parts of northern China. It suggests that wheat spread to the northern steppe first and then went southwards into different parts of the Yellow river region along various parallel routes (Zhao 2009; Barton and An 2014; Zhao 2015b). A third hypothesis attempts to grapple with the apparently earliest occurrence of wheat in eastern China, on the Shandong peninsula (Jin et al. 2011; Long et a; 2018; Liu et al. 2019) and shortly thereafter in northern Fujian (although without direct dating, Jiao 2007, p. 246), suggesting even the possibility of coming by sea from India (Zhao 2012; Liu et al. 2019), or perhaps via the easternmost Mongolian steppe and via northeastern China to reach Shandong first. Long et al. (2018) have argued for trading in wheat as a luxury item via the eastern Mongolian steppe initially to the lower Yellow river region (Shandong). Obviously, reliable dates of the first appearance of wheat in each region would be central to determining the likelihood of these various models. The present paper focuses on central China and the region *Zhongyuan*, which includes the central part of Shaanxi, Shanxi and Henan provinces. This was the core region in which urbanization, starting at the end of the Longshan period around 4,000 years ago, led to the development of the first recognized Chinese state in the early Bronze Age, the Erlitou culture, and subsequently the Shang dynasty.

However, most direct radiocarbon dates on wheat grains at present are from northwest China, while there are only a few from central and eastern China. This situation has been even worse in central China, as all sites with reported late Neolithic wheat remains have had their dates estimated from archaeological evidence such as pottery typology instead of direct dating. The only site with directly dated late Neolithic wheat grains yielded a result much later than 2000 bc (Zhao 2015b). From eastern China, only three AMS dates from wheat older than 2000 bc have been reported, and the oldest one is from Zhaojiazhuang with a 2-σ calibrated age of 2570–2200 bc (Jin et al. 2011; Long et al. 2018). From northwest China only four direct AMS dates are just before 2000 bc, but have a higher probability of falling between 2000 and 1800 bc and come from Bronze Age cultural contexts that are expected to be early 2nd millennium bc (Dodson et al. 2013, Table 1). Despite some claims that wheat might have entered China ca. 3000 bc (for example, Li et al. 2007; Dodson et al. 2013), the empirical evidence is now strongly against this, with the bulk of well-dated wheat grains from after 2000 bc (Zhao 2015b; Liu et al. 2016; Stevens et al. 2016). This raises the question as to how much before 2000 bc, if at all, wheat was present in China, in which areas, and how significant it was in agriculture and diet at that time, as well as when it rose to prominence. Ancient Chinese written sources indicate that wheat was known, and was perhaps something of a delicacy, but still a minor crop, from the late Bronze Age (Bray 1984), and thereafter it became increasingly important as a staple food for the poor throughout the Han dynasty, before later becoming more favoured as new flour-based foods emerged over the course of this period, 202 bc-ad 263 (Yü 1977).

**Table 1:** Direct AMS radiocarbon dates on cereal grains from Xiazhai and three other sites

Site name	Lab code	Sample type	Context no.	Cultural period	^14^C date (uncal bp)	Cal age (2σ-range)
Xiazhai	BA151801	Wheat grain	G39	Han dynasty^a^	1,955 ± 20	19 bc-ad 118
	BA151800	Wheat grain	H1411	Han dynasty^a^	2,200 ± 20	360-200 bc
	BA151799	Wheat grain	H652	Eastern Zhou^a^	2,320 ± 20	406-374 bc
	BA151798	Wheat grain	H1099	Eastern Zhou^a^	2,515 ± 20	786-547 bc
	BA151797	Wheat grain	H209	Late Longshan^b^	2,510 ± 25	789-542 bc
	BA151796	Wheat grain	H255	Late Longshan^b^	2,415 ± 30	746-401 bc
	BA151795	Wheat grain	H2850	Shijiahe^b^	2,490 ± 25	774-524 bc
	BA151794	Wheat grain	H2773	Shijiahe^b^	2,345 ± 25	486-377 bc
	BA151793	Rice grain	H2497	Shijiahe^a^	3,915 ± 35	2487-2291 bc
	BA151792	Wheat grain	H2497	Shijiahe^b^	2,250 ± 20	391-209 bc
	BA151791	Foxtail millet grains	H2247	Yangshao^a^	4,585 ± 25	3496-3127 bc
Baligang	UGAMS#27671	Wheat grain	H1884	Late Longshan^a^	3,670 ± 25	2137-1966 bc
Baligang^c^	BA081053	Rice grain	H1880	Eastern Zhou^a^	2,560 ± 35	810-540 bc
Baligang^c^	BA081054	Foxtail millet grains	H1880	Western Zhou^a^	2,935 ± 35	1230-1010 bc
Baligang^c^	BA081055	Wheat grain	H1880	Eastern Zhou^a^	2,500 ± 35	790-510 bc
Nanjie	UGAMS#27672	Wheat grain		Late Longshan^b^	100 ± 20	ad 1691-1925
Nangao	UGAMS#27673	Wheat grain		Late Longshan^b^	1,580 ± 20	ad 421-539

Samples with lab codes beginning BA were all dated in the laboratory for accelerator mass spectrometry (AMS) radiocarbon dating at Peking University; samples with lab codes beginning UGAMS were all dated in Center for Applied Isotope Studies, The University of Georgia

^a^Cultural period, based on stratigraphy, matches the radiocarbon age

^b^Grains intrusive

^c^Deng et al. (2015)

In central China, an accurate dating of the appearance of wheat and its importance or frequency relative to other contemporary crops would be of great significance for our understanding of the economic foundations of ancient central Chinese civilization. Based on the early arrival of wheat in northern China, it is argued that a multi-crop system of *Wugu* (five main crops), comprising *Setaria italica*, *Panicum miliaceum*, *Triticum aestivum*, *Oryza sativa* (rice) and *Glycine max* (soybean), had already been established there in the late Longshan period (ca. 2300–1800 bc) (Liu et al. 2015, p. 332). A further idea is that this polyculture farming strategy had encouraged the rise of ancient civilization in central China, because a multiple crop system is more reliable for the stable development of ancient societies, compared to the rice-based monoculture system in southern China, especially when facing natural hazards due to the inter-annual variability in productivity of any one crop (Zhao 2011).

Obviously, the uncertainty about the date of the first appearance of wheat in central China has not only started the debate on the route of wheat diffusion into China, but it has also restricted our understanding of the significance of exotic food elements in the rise of ancient Chinese civilization. Previous studies have shown that stratigraphic intrusion, in which later remains have become embedded in earlier layers, is a significant factor when dealing with archaeobotanical records, especially for studies of the first appearance of specific crop types in certain regions (Pelling et al. 2015; Liu et al. 2017). A study based on direct AMS radiocarbon dating of *Panicum* grains has proved that millet actually spread into Europe much later than had been previously assumed (Motuzaite-Matuzeviciute et al. 2013), around 1500 bc rather than before 5000 bc, which had sometimes been inferred from the archaeological contexts (Hunt et al. 2008; Valamoti 2016). A review of British AMS dating results on crops also shows that a large proportion, as many as ca. 17% of predicted Neolithic or Bronze Age crop remains, actually proved to be much later, Iron Age or more recent still (Stevens and Fuller 2012), while a similar estimate for Ireland was at least 10% (Whitehouse et al. 2014). Therefore, direct dates from ancient wheat grains should provide fundamental information from which to begin an assessment of the actual role of wheat in the subsistence systems of late Neolithic central China.

**Fig. 1:**
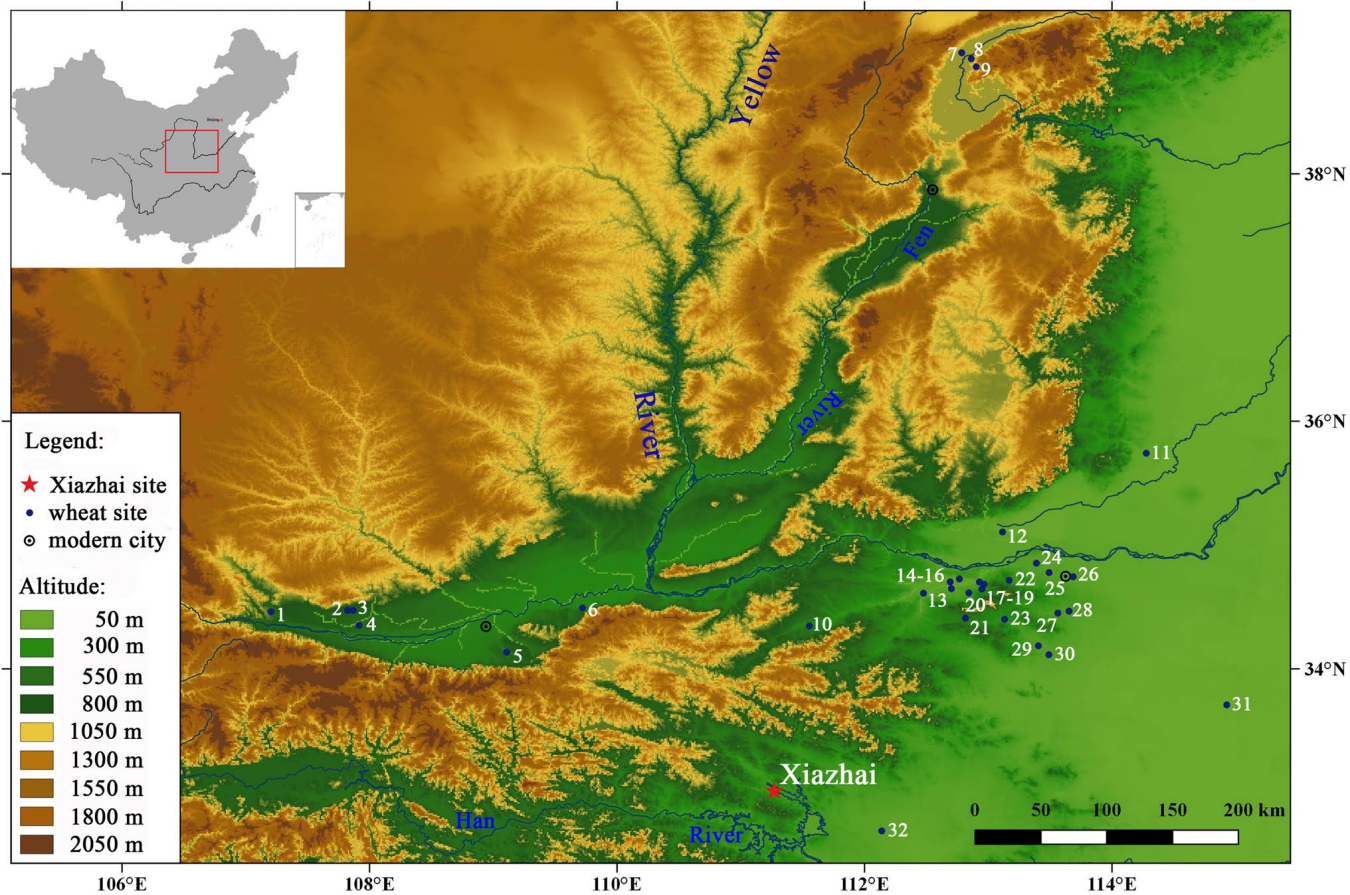
Location of Xiazhai and other Neolithic and Bronze age sites with wheat remains in central China. *1* Shangguancun, *2* Wangjiazui, *3* Zhuangli, *4* Anban, *5* Donggao, *6* Nansha, *7* Nangao, *8* Nanjie, *9* Baishi, *10* Hucun, *11* Shuinan, *12* Jingyanggang, *13* Dalaidian, *14* Xijincheng, *15* Zaojiaoshu, *16*–*18* Gaoya, Erlitou, Yanshishangcheng, *19*–*21* Shaochai, Feiyaonan, Tianposhuiku, *22* Matun, *23* Nanwa, *24* Fengzhai, *25* Wangchenggang, *26* Guanzhuang, *27* Dongzhao, *28* Zhengzhoushangcheng, *29* Xinzhai, *30* Guchengzhai, *31* Wadian, *32* Dugangsi, *33* Pingliangtai, *34* Baligang. Map created in QGIS2.18.12, https://www.qgis.org. Base map is from SRTM 90 m Digital Elevation Data, http://www.gscloud.cn/

Towards this end, the present paper reports the results of systematic archaeobotanical work at the Xiazhai site in central China and direct dates on wheat grains from there and from another three sites in central China (Fig. 1). Together with direct dates on late Neolithic wheat grains from some other sites elsewhere in China and a review of early wheat reports without direct dates, we offer an updated assessment of the timing of wheat adoption in central China and its role in the latest Neolithic to early Bronze Age there.

## Materials and methods

### Xiazhai site description

The Xiazhai site (111°16′13″E, 33°01′01″N) is located in the southwest part of the Nanyang basin, Henan Province (Fig. 1). Large quantities of archaeological remains have been recovered through years of excavations there from 2009 to 2013 (Chu and Cao 2011). According to these remains, this site was first occupied in the Yangshao period (ca. 4300–3000 bc) and then after a roughly 500 year hiatus, it continued to be used into the Shijiahe (ca. 2500–2300 bc) and late Longshan periods (ca. 2300–1800 bc). Deposits above these Neolithic remains belong to the Erlitou culture period (ca. 1800–1500 bc), Western Zhou dynasty (1046–771 bc), Eastern Zhou dynasty (770–221 bc) and the later historical periods from the Han to Qing dynasties (202 bc-ad 1911). Various kinds of features, such as storage pits (secondarily refilled with occupational refuse), houses, a moat, burials and kilns were discovered from different periods at this site, providing a very rich archaeological sequence.

### Collection of plant macro remains

Flotation samples for archaeobotanical analysis were collected from 690 contexts from different periods, during excavation at the Xiazhai site. 7,000 l of soil samples were floated at the site by the wash-over bucket flotation method (Pearsall 2000, pp. 29–44; Fuller 2008). Plant macro remains were collected on sieves with a smallest mesh size of 0.3 mm, air dried and then further studied in a laboratory. All samples were weighed and measured before being sorted under a binocular stereomicroscope. Seeds, fruits and other parts of plants were separated from wood charcoal and identified with reference to modern collections and atlases of fruits and weeds in China (Wang 1990; Guo 2009), as well as previously reported archaeobotanical evidence (ESM 1).

### AMS radiocarbon dating

Eleven cereal grain samples from the Xiazhai site were sent to the National Key Laboratory for accelerator mass spectrometry (AMS) radiocarbon dating at Peking University. Nine dated samples were single wheat grains from different cultural contexts, and one rice grain from the same Shijiahe context as a wheat grain was also dated for comparison. In addition, five *Setaria* (foxtail millet) grains from a Yangshao context were combined as a single sample for dating to ensure enough carbon after pre-treatment.

In order to further address the date of wheat adoption in central China, three more charred wheat grains from other sites were sent to the Center for Applied Isotope Studies, The University of Georgia, USA, for direct AMS radiocarbon dating. One was from Baligang (Deng et al. 2015; Weisskopf et al. 2015), a site near Xiazhai, the other two were from the Nangao and Guojiazhuang sites in the northern part of Shanxi (Jiang 2017).

## Results

### AMS radiocarbon dates

All samples yielded radiocarbon dates successfully. Each date was calibrated by OxCal v4.2.4 (Bronk Ramsey 2013), using the IntCal13 atmospheric curve (Reimer et al. 2013), and they are presented in Table 1. Of 14 submitted radiocarbon samples, half came back as apparently intrusive, including six out of seven wheat grains which were thought to derive from 3rd millennium bc contexts, Late Longshan or Shijiahe periods. Only a single wheat grain was dated to before 2000 bc, from a Late Longshan period context at Baligang (archaeobotanical data, Deng et al. 2015; Weisskopf et al. 2015).

The Yangshao millet sample from Xiazhai is dated to 3496–3127 bc (95.4% probability), which fits well with the date estimated from its archaeological context. For the Shijiahe samples, the charred rice grain from the context H2497 yielded a date calibrated to 2487–2291 bc, also in agreement with the associated pottery assemblage. However, the measured date of the charred wheat grain from the same context was only 391-209 bc. Similarly, the other four wheat grains from Shijiahe and late Longshan period contexts produced late Bronze Age dates, instead of the predicted late Neolithic age of around 2000 bc. By contrast, most of the Eastern Zhou and Han wheat samples yielded reasonable radiocarbon dates in accordance with their associated archaeological contexts.

The Baligang wheat sample yielded a date of 2137–1966 bc (95.4% probability), just falling within the expected late Longshan period. However, as with the Xiazhai samples, the other two wheat grains from the northern part of Shanxi Province, which were dated to ad 1691–1925 and ad 421–539 respectively, both proved to be later intrusions from the historical period or even modern times.

In previous studies, direct radiocarbon dating on wheat has been carried out at ten late Neolithic and Bronze Age sites and 18 direct radiocarbon dates of early wheat from nine sites have been published from central China (ESM 2). Previously, only the Wadian site had included direct radiocarbon dating on supposedly late Neolithic wheat remains, which produced several dates of the historical period, but the original data have not been published (Zhao 2015b). From the following Erlitou period, no direct radiocarbon dates on wheat have been obtained. Archaeological sites of the Shang dynasty have produced the largest number of direct dates so far, and most of these are in accord with the age of the archaeological context, roughly extending from 1600 to 1000 bc. Three dates from the Western Zhou and Eastern Zhou periods were also obtained from samples from Baligang, Wangchenggang and Dong’gao. Even at the Bronze Age sites, obvious evidence of intrusion can still be found, such as the historical dates obtained from apparent Shang dynasty levels at the Shangguancun site and the Western Zhou levels at Donggao (ESM 2).

### Crop assemblages in different periods at the Xiazhai site

**Fig. 2:**
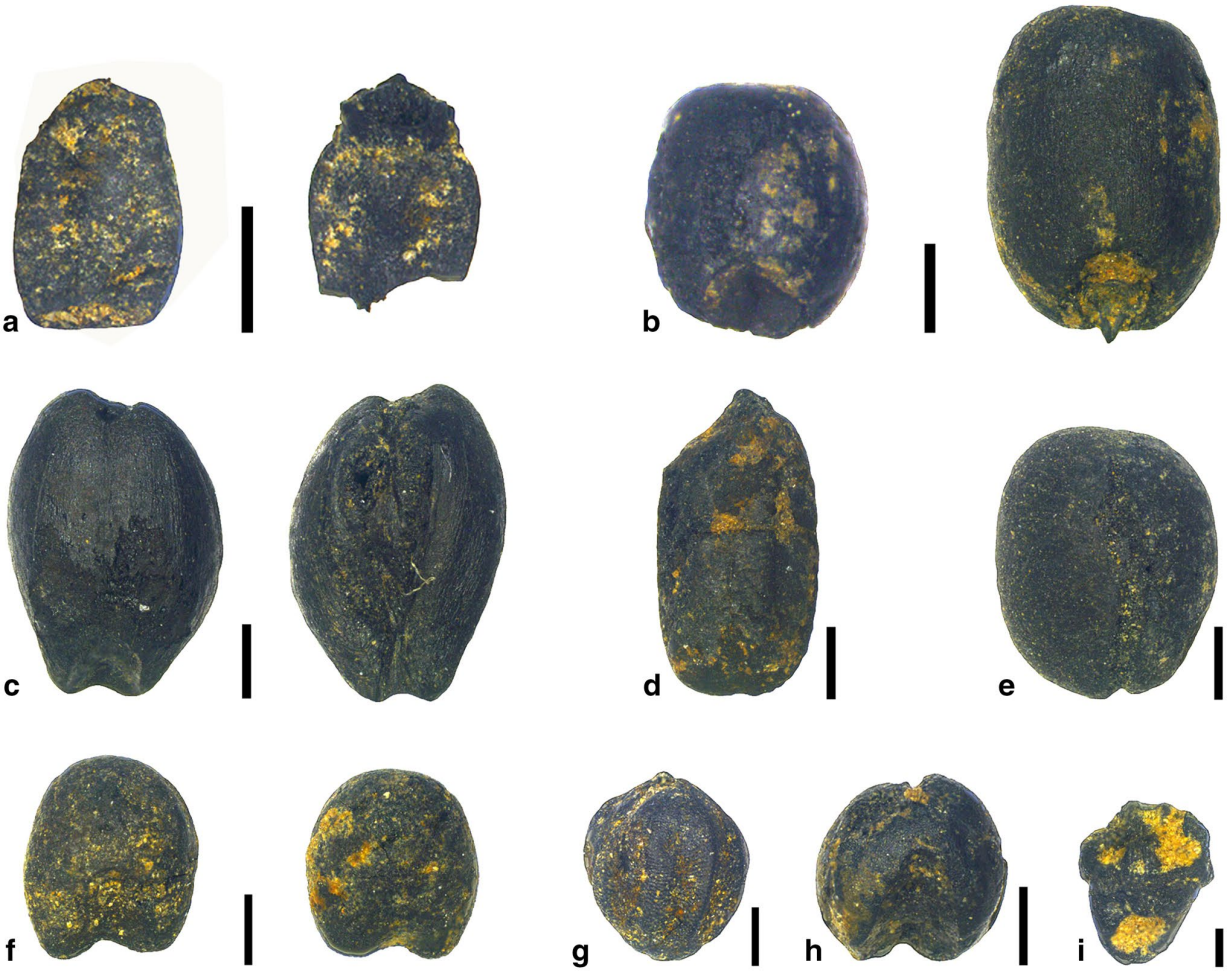
Charred remains of representative crops from the Xiazhai site. **a** wheat rachis of hexaploid type (*Triticum aestivum*); **b***Triticum sp.* grain of free-threshing type; **c***Hordeum vulgare*; **d***Oryza sativa*; **e***Glycine max*; **f***Panicum miliaceum*; **g***Setaria italica* with husk; **h***Setaria italica*; **i***Oryza* spikelet base. Scale bar **a**–**e**, 1 mm; **f**–**h**, 500 μm; **i**, 200 μm

7,920 seeds from more than 38 taxa were identified in 690 samples, comprising cereals, fruits and field weeds (ESM 1). Cereals were dominant among all plants identified from Xiazhai, making up 88.2% of the total count. *Oryza sativa*, *Setaria italica*, *Panicum miliaceum*, *Triticum* cf. *aestivum*, *Hordeum vulgare* (barley) and *Glycine max/soja* were the six types of crops found here (Fig. 2).

**Fig. 3:**
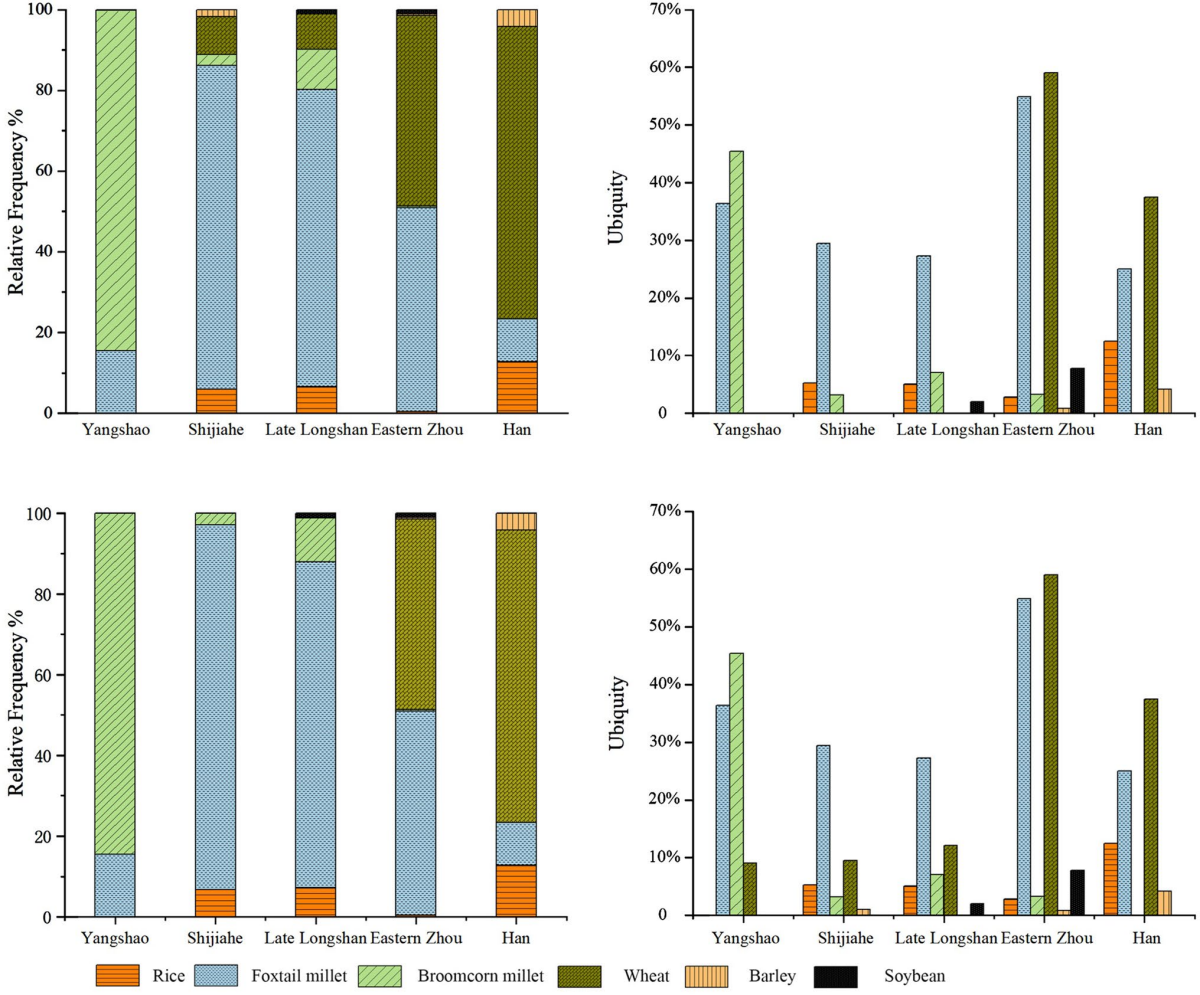
Comparison of relative frequencies and ubiquities of different crops in all periods at the Xiazhai site: **a** and **b**, relative frequencies and ubiquities based on original data; **c** and **d**, relative frequencies and ubiquities based on data with intrusions excluded

All floated samples from the Xiazhai site can be divided into one of five periods, including Yangshao, Shijiahe, late Longshan, Eastern Zhou and Han dynasty, according to their archaeological context (ESM 1). *S. italica* and *P. miliaceum* were the two main crops found in the Yangshao samples (Fig. 3a, b). One charred wheat grain was also found in a sample of this period, but it is presumed to be intrusive.

In the Shijiahe period, *S. italica* was the most important crop at Xiazhai, as demonstrated by its frequency (proportion or percentage of total crop remains) and ubiquity (number of samples in which it occurred) (Fig. 3a, b). Eleven wheat grains were found in nine samples of this period. The frequency and ubiquity of wheat in the Shijiahe samples appear even higher than those of contemporary *O. sativa* and *P. miliaceum* (Fig. 3a, b). These wheat grains were all of the plump free-threshing type, while three preserved rachis segments were hexaploid (*T. aestivum*) type. In addition, two charred barley grains were also found in one sample. A similar crop assemblage also occurred from the late Longshan period (Fig. 3a, b). A possible change from the late Longshan period is the addition of soybean, but only two seeds were found in two different contexts. Taking into account the likelihood that wheat and barley may have been intrusive in levels older than ca. 2000 bc, based on the radiocarbon results from this site, the statistics of crop presence were recalculated without wheat and barley in these older deposits, but with little apparent effect (Fig. 3c, d).

During the Eastern Zhou dynasty, *S. italica* and *Triticum* were the two staple crops at the Xiazhai site, as revealed by both frequencies and ubiquities of all crops (Fig. 3a, b). Wheat almost acquired a similar status to native *S. italica* in the subsistence of Eastern Zhou people there. *Oryza sativa*, *P. miliaceum*, *Hordeum* and *G. max* still appear in small numbers. During the following 500 years, the degree of dependence on wheat grew very quickly. During the Han dynasty, both its frequency and ubiquity were much higher than *S. italica*, which had been the main staple crop at the Xiazhai site for nearly 4,000 years (Fig. 3a, b).

### Previous finds of early wheat in central China

According to the published data, wheat macro remains have been collected from 14 sites of the Longshan period in Henan, Shanxi or Shaanxi (ESM 2). Nevertheless, the total number of finds is quite limited. Baligang and Wangchenggang are the two sites that produced the greatest number of wheat remains from this period, from which nine and eight wheat grains have been found respectively (Zhao and Fang 2007; Deng et al. 2015), but only one or two specimens have been found in other sites. Accordingly, the ubiquities of wheat at these sites are all quite low, generally less than 5%. Similar rarity could be observed in the following Erlitou period, as indicated by archaeobotanical data from six sites (ESM 2).

In contrast to the sparse discoveries from the previous periods, a considerable number of wheat remains have been recovered from each site of the Shang dynasty (ESM 2). At present, archaeobotanical work has been done at five sites of the early Shang period and four of the late Shang in central China. Except for the Erlitou, Nanwa and Guchengzhai sites, 60 or even more wheat grains have been recovered from each of the other sites (Chen et al. 2012; Wu et al. 2014; Zhao 2015a). Ubiquities of wheat at most sites are also much higher than from the Longshan and Erlitou periods (ESM 2), and the radiocarbon dates on seeds accord with the Shang cultural period (ESM 2).

The rising trend of wheat in crop systems continued in the Western and Eastern Zhou periods. Meanwhile, some distinctions between different sites of the same period start to appear. At the Zhuangli site, 533 wheat grains were recovered from 18 samples of the Western Zhou dynasty and the ubiquity reached 94.44% (Zhouyuan Archaeological Team 2011). By contrast, some sites like Anban, Dongzhao and Wangjiazui produced no wheat (Zhao and Xu 2004; Liu 2014; Yang et al. 2017).

In addition to these finds from systematic archaeobotanical research at excavated sites, some occasional discoveries of wheat remains are also reported from additional small scale sampling during regional surveys (ESM 2). From the field survey of the Yiluo river valley, wheat remains have been reported from two sites of the Shang dynasty, namely Fengzhai and Tianposhuiku, each of which produced 92 and 172 wheat grains respectively (Lee et al. 2007). In the archaeobotanical survey of the Luoyang basin, wheat remains were reported from one site of the Longshan period, two of the Erlitou period, one of the Shang dynasty and one of the Eastern Zhou dynasty, from each of which less than ten wheat grains were recovered (Zhang et al. 2014). In the Ying valley, only one sample of the Shang dynasty from the Dugangsi site yielded two wheat grains as well some barley (Fuller and Zhang 2007). Three sites of the Longshan period in the Hutuo river valley in Shanxi Province also produced many wheat remains (Jiang 2017). In the Sushui survey two sites produced wheat associated with Longshan finds, from each a single grain, but from Shuinan there were also three hexaploid wheat rachis segments, in contrast to the 485 millet remains in the same sample (Song et al. 2019). Wheats were also reported from two surveyed Bronze Age sites in Shaanxi Province, but the total amount of the discovery was not reported (Dodson et al. 2013).

The archaeobotanical data from excavated sites and regional surveys are mostly in accordance with each other. Most Longshan and Erlitou sites only yielded one or two wheat grains. Since the Shang period, larger numbers of wheat grains, for instance 100 grains or even more, begin to appear at a few sites. However, this varies greatly between different sites, with many sites still lacking wheat. Therefore wheat was possibly not very common during the Shang dynasty (ca. 1600–1046 bc), as suggested by samples from regional surveys, but it then became more common during the Zhou dynasty (1046–256 bc).

## Discussion

### The appearance of wheat at the Xiazhai site

According to the excavated contexts and the associated archaeological finds, the earliest wheat at the Xiazhai site is from the Yangshao context H1086. However, the time period of the Yangshao culture is at least 1,000 years earlier than the earliest wheat grains discovered in China anywhere else, so far (Dodson et al. 2013; Liu et al. 2016; Long et al. 2018). Therefore, this wheat grain is likely to represent intrusive material, which was worked downwards by bioturbation.

From later periods, several wheat grains have been recovered from Shijiahe and late Longshan contexts. Nine Shijiahe contexts and 12 late Longshan contexts yielded 27 charred wheat grains, but in most cases only a single or at most two grains of wheat were found in each case, and in all cases these represent a small trace presence compared to the dominant foxtail millet remains in these samples. Based on the archaeological context, the ages of these wheats would be expected to be between 2500 and 1800 bc, which is indeed the period when wheat seems to have first been introduced to China (for example, Flad et al. 2010; Jin et al. 2011; Betts et al. 2013; Dodson et al. 2013; Liu et al. 2016; 2019; Long et al. 2018). There is also other evidence for cultural exchanges between China and Central Asia, including the introduction of livestock towards the beginning of this period and metallurgy towards the end, as well as the transfer of various crops of Chinese origins to Central Asia, India and beyond (for example, Stevens et al. 2016). However, direct radiocarbon dating results of five wheat grains from the Shijiahe and late Longshan periods reveal that the oldest directly measured date is around 780 bc, so this is intrusive material from the Eastern Zhou period. Longshan period wheats from Guojiazhuang and Nangao proved to be intrusive from even later periods. Moreover, a comparison between direct dates of rice and wheat from the same Shijiahe context H2497 also confirms the possibility of later wheat intrusion, as the date of this charred rice fits well with its archaeological context, while the date of the wheat is more than 2,000 years later than that (Fig. 2; Table 1). Obviously, a contradiction between the direct dates of ancient wheat grains and their context ages estimated by archaeological finds is a recurrent problem, with all of the possible Shijiahe and late Longshan wheat remains probably being later intrusions, with the exception of a single case from Baligang, which we shall return to below.

The direct dating results of wheat grains from the Eastern Zhou and Han dynasties are, however, normally consistent with their cultural context ages. Hence, current evidence demonstrates that wheat first appeared at Xiazhai during the Eastern Zhou dynasty. The apparently earlier undated wheat remains from Shijiahe and late Longshan contexts are therefore likely to represent later contamination from the Eastern Zhou period or later. This suggests that once wheat became a prominent part of subsistence in the later Bronze Age, intrusive charred grains worked their way down into earlier deposits through processes such as bioturbation.

By eliminating these later intrusive wheat grains, a different pattern of crop assemblage change can be reconstructed for the Xiazhai site. From the Yangshao to the late Longshan period, millets, especially *Setari*a (foxtail millet), were dominant as the daily staples of the people there, along with a small portion of later introduced rice and soybean (Fig. 3c, d). In the Eastern Zhou and Han dynasties, wheat acquired equal importance in the food assemblage with foxtail millet, and even slightly exceeded the latter in the Han dynasty (Fig. 3c, d). Given the fact that wheat reached such a high proportion of all crops at the Xiazhai site just when it first appeared in the Eastern Zhou period, it is quite possible that wheat actually entered the southwest part of Henan Province earlier than the Eastern Zhou dynasty. This speculation is consistent with the newly dated wheat grain from the Baligang site, as well as previously dated evidence from the Western Zhou period (Deng et al. 2015).

### Re-evaluating the role of wheat in late Neolithic central China

Besides the Xiazhai site, wheat has been reported from more than 17 Neolithic and Bronze Age sites in central China (Fig. 1, ESM 2). According to the age of the archaeological contexts, only eight sites have yielded wheat grains from the late Longshan period in Shanxi, Shaanxi or Henan provinces (Zhao and Xu 2004; Chen et al. 2010; Liu and Fang 2010; Zhao 2011; Liu 2014; Deng et al. 2015; Zhong et al. 2016). However, most of these late Neolithic wheat grains have not been directly dated. The newly dated wheat from Baligang in this study is the only late Neolithic wheat from central China which is supported by a direct date. Besides, carbonized wheat grains from the Wadian site were also sent for radiocarbon dating, but the results turned out to be much later than Longshan period (Zhao 2015b). Another noteworthy point is that these early wheat grains are all very few in number (ESM 2). Only 24 possibly late Neolithic wheat grains have been reported from the other six sites in central China, and eight of them from the Wadian site have been proven by direct dating to be later intrusions (Zhao 2015b). None are from the actual Neolithic period. Outside central China, there are three direct AMS dates on wheat from Longshan period contexts at Dinggong and Zhaojiazjang in Shandong near the lower Yellow river (Long et al. 2018). At Zhaojiazhuang, wheat was represented by ten grains, just over 1% of the identified crops, with *Oryza* and *Setaria* accounting for most of the other crop remains (Jin et al. 2011). Also in Shandong, just two wheat grains were found in an assemblage of over 5,000 seeds at the Late Longshan site of Liangchengzhen (2200–1850 bc), but these have not yet been directly dated (Crawford et al. 2005). Long et al. (2018) also report intrusive wheat grains based on direct dates from four other sites in Shandong. In other words, Shandong has a similar pattern to that in central China, with only three confirmed wheat occurrences older than 2000 bc and rather more (seven) that have been shown to be intrusive in deposits of that age (Long et al. 2018).

Discoveries of wheat from the Early Bronze Age, the Erlitou period, are still quite limited. Even at the Erlitou site, which is believed to be the central site of this region, only three wheat grains were found in the latest phase of this culture (Zhao 2015a). From the following Shang dynasty, wheat grains have been found at some sites in considerable quantity, such as at Wangchenggang, Fengzhai, Tianposhuiku in Henan Province and Zhouyuan in Shaanxi Province (Zhao and Xu 2004; Lee et al. 2007; Zhao and Fang 2007). Direct radiocarbon dating results also confirm the presence of wheat since ca. 1700 bc in central China. However, in these Bronze Age sites with direct dates of wheat grains, obviously intrusive materials from later historical periods can still be observed (for example, Dodson et al. 2013; Liu et al. 2016).

Overall, the direct date on one wheat grain from Baligang in this study produced the only wheat which was actually Neolithic, around 2000 bc in central China, while other dated wheat grains are all later than 1700 bc. The total quantity of possible late Neolithic wheat is no more than 20 or fewer grains. Given the fact that intrusions into earlier layers by early wheat remains appear to have been quite common on many sites, we conclude that wheat was both rare and played an unimportant role in the agricultural systems of late Neolithic central China.

The limited role of wheat in the late Neolithic diet agrees with stable isotope data from human skeletons where they are available. Although staple isotopes cannot determine the species consumed, in the context of northern China the role in the diet of millets, which are C_4_ plants can be inferred, in contrast to C_3_ plants, which include wheat as well as rice, soybean and many others (for example, Lightfoot et al. 2013). The Longshan assemblages, like those of Yangshao period, have a strongly C_4_ diet signature (Chen et al. 2016). Assemblages from Eastern Zhou, by contrast, are significantly different, with a larger C_3_ plant contribution that suggests consumption of significant quantities of wheat (Dong et al. 2017a, b).

**Fig. 4:**
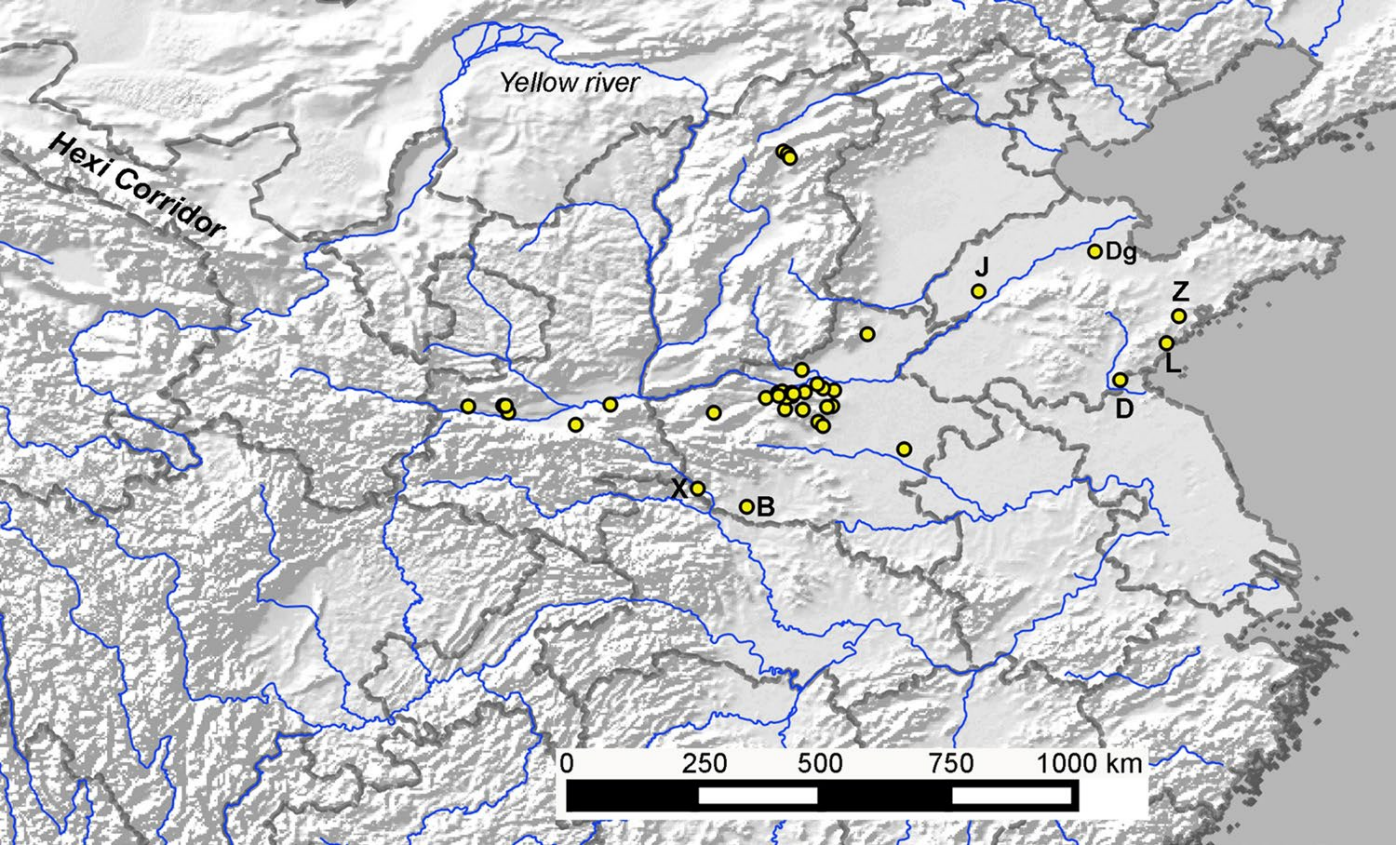
Map of sites with early wheat finds in central China shown in relation to reported early wheat finds from the Shandong and Hexi corridor region. Selected sites are labelled *X* Xiazhai, *B* Baligang, *Z* Zhaojiazhuang, *L* Liangchenzheng, *J* Jiaochangpu, *D* Dongpan, *Dg* Dinggong. For other site names, see Fig. 1

In light of empirical dating evidence, it may be worth reconsidering the route by which wheat arrived in central China. Currently there is an absence of pre-2000 bc wheat evidence in northern Shanxi, Shaanxi or Inner Mongolia. Evidence from northwest China includes some wheat finds from around the start of the 2nd millennium bc (Dodson et al. 2013). The site of Baligang produced a direct date as early as or earlier than the earliest age of wheat from the Hexi corridor. In eastern China, the direct dating results from the Zhaojiazhuang and Dinggong sites in Shandong Province suggest the presence of a small of amount of wheat there by ca. 2200 bc (Jin et al. 2011; Long et al. 2018). There are additional reports of a few wheat grains associated with Longshan cultural contexts in Shandong from Liangchenzheng (Fig. 4; Crawford et al. 2005) and Jiaochangpu (Zhao 2014), although these might be intrusive grains in the absence of direct dates, as evident at Dongpan (Wang et al. 2011; Long et al. 2018). The age of wheat from Longshan contexts at Shandong is similar to that of the earliest dated wheat in eastern parts of Central Asia, ca. 2460–2150 bc in Kazakhstan (Frachetti et al. 2010; Doumani et al. 2015; Spengler et al. 2016), which would fit with the idea that wheat first arrived as part of long distance trade in luxuries with distant Central Asian populations, through intermediaries in the Mongolian region (Long et al. 2018). There are neither clear indications of the route by which wheat entered China at present, nor whether this might have followed more than one route, although the Silk Road pathway from Central Asia via Xinjiang appears to be less likely. Nevertheless, as noted by Barton and An (2014), it is to be expected that the initial arrival of an innovation such as a new crop would be elusive. The few apparently late Longshan context wheat occurrences from Shandong mentioned by Long et al. (2018) could represent the same arrival through trade as that shown by the dated example from Baligang in southern Henan.

### The role of wheat in Bronze Age agricultural systems of central China

As discussed above, any cultivation or consumption of wheat at this time was at best sporadic and it clearly did not form an important part of the cropping system in central China in the late Neolithic period. The emergence of complex societies in the Longshan period certainly had nothing to do with the introduction of exotic crops and the related *Wugu* multi-crop system. A thorough review of all the archaeobotanical data also clearly illustrates a farming system in the Longshan period in central China which was mainly based on *S. italica* as the staple grain, together with a small amount of *P. miliaceum*, *Oryza* and *Glycine* cultivation (Deng and Qin 2017; Stevens and Fuller 2017). Additional crop diversity may have come from oilseeds or herbs like *Cannabis sativa*, *Perilla frutescens* and *Brassica juncea,* as well as some possible fruit trees, such as *Ziziphus jujuba, Armeniaca vulgaris*, *Prunus persica* and *Morus alba* (Fuller and Stevens 2019). This cropping system can be traced back to the Yangshao period, and the same range of crops was quite common at late Neolithic sites in other regions (Lee et al. 2007; d’Alpoim Guedes 2011; Deng 2013; Jin 2013; Liu 2014; Stevens and Fuller 2017; Deng et al. 2018). In other words, the rise of complex societies in central China was based on the traditional millet farming of the northern Chinese Neolithic, and there is nothing agriculturally distinctive, such as new cereals, that suggest economic superiority there compared with contemporary agricultural systems in adjacent regions.

**Fig. 5:**
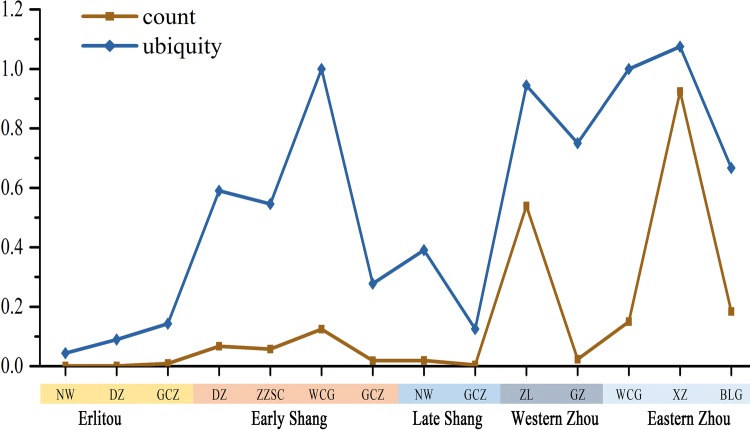
Ratio of counts and ubiquities of wheat and foxtail millet (wheat/foxtail millet) of Bronze Age sites in central China. *NW* Nanwa, *DZ* Dongzhao, *GCZ* Guchengzhai, *ZZSC* Zhengzhoushangcheng, *WCG* Wangchenggang, *ZL* Zhuangli, *GZ* Guanzhuang, *XZ* Xiazhai

Nevertheless, since current evidence does confirm the introduction of wheat into central China in the early Bronze Age, it is necessary to clarify its status in the agricultural systems there in that period and its role in the development of early states in China. For this purpose, we have considered the quantitative comparison of wheat and foxtail millet ratios for each site at which wheat occurred, both in terms of relative frequency (proportion) and ubiquity, and also by date (Fig. 5). For frequency, the number of wheat grains found at each site is normally less than 10% of the *S. italica* number, except for Zhuangli and Xiazhai. The ubiquity generally continues rising from the Shang dynasty period throughout the Bronze Age, although it varies between different parts of northern China. The ubiquities of wheat from Nanwa and Guchengzhai are very low in all periods, while those of the Wangchenggang site are always much higher than other sites. In addition, one must keep in mind that the majority of sites with archaeobotanical evidence have no evidence for wheat (Lee et al. 2007; Zhang et al. 2014; Jiang 2017).

On the whole, we think that people did not depend much upon wheat, but it increased in importance amongst some communities in the Later Bronze Age, from the Western Zhou period onwards (after ca. 1000 bc). A steady increase can perhaps be traced since the Shang dynasty (after ca. 1600 bc), as shown by the ubiquities. This is supported by stable isotope analyses of human bones from many Bronze Age sites in this region. The value of δ^13^C changes from roughly − 7‰ at the Niedian site of the Erlitou period, a strongly C_4_ signal, to almost − 13‰ at the Shenmingpu site of the Eastern Zhou period, revealing a clear increase of C_3_ food in the routine consumption of local people (Hou et al. 2012; Wang et al. 2014). Meanwhile, as with the ubiquity values, the δ^13^C values from many other Bronze Age sites vary greatly between − 7 and − 13‰ (Zhou and Garvie-Lok 2015). For instance, the value from the Eastern Zhou site of Jianhe is only between − 8  and − 10‰, showing strong dependence on millets (Ling et al. 2011). The C_3_ contribution has recently been shown to be even greater among female and low status burials around the city of Zheng Han during the Eastern Zhou period (Dong et al. 2017a, b), which supports the suggestion that when wheat increased in agricultural importance, it was associated with providing more calories for those of lower status (Yü 1977; Boivin et al. 2012).

Integrating archaeobotanical data with stable isotope data from human bones, it can be confirmed that wheat was not of great significance in the overall subsistence system, but it did increase gradually throughout the Bronze Age in central China. Even in the late Bronze Age, many sites still relied on traditional millets, especially *S. italica*, for their daily staples instead of a mixture of millets and wheat.

## Conclusion

The Xiazhai site yielded the largest number of wheat grains from late Neolithic contexts so far found at any site in central China, but when radiocarbon dated they all proved to be the probable results of intrusion of later grains into earlier levels. This highlights that the importance of wheat in the late Neolithic has been over-estimated, and that wheat was even rarer than has been supposed. Nevertheless, the direct date on one wheat grain from the Baligang site, as with the three published dates on grains from two sites in Shandong (Jin et al. 2011; Long et al. 2018), confirm that wheat had arrived in central China at least by around 2000 bc. The recurrence of intrusive grains in earlier contexts and the limited quantity of wheat dated to the Neolithic from all sites suggest that wheat was of little significance in the subsistence strategy of late Neolithic people living in central China, and it was not central to the Bronze Age agriculture upon which early Chinese states relied.

## Electronic supplementary material

Below is the link to the electronic supplementary material.

Supplementary material 1 (XLSX 204 kb)

Supplementary material 2 (DOCX 23 kb)
