# Supplementary material for: Assessing the occurrence and status of wheat in late Neolithic central China: the importance of direct AMS radiocarbon dates from Xiazhai
Source: Veg Hist Archaeobot. 2019 Jun 3;29(1):61–73. doi: 10.1007/s00334-019-00732-7 (PMC6942569; doi:10.1007/s00334-019-00732-7)
Supplement: Supplementary file 2 — Supplementary material 2 (DOCX 23 kb) [file 334_2019_732_MOESM2_ESM.docx]

**TABLE S2. Archaeological sites in Central Chinese provinces with reported finds of wheat, and direct AMS radiocarbon dates on wheat.** Site names followed with * are survey sites with small-scale sampling, others are large scale excavations.

| Site name | Region | Longshan^1^ | | Erlitou | | Shang | | Western Zhou | | Eastern Zhou | | Wheat AMS Radiocabon Lab code | Uncalibrated C-14 date BP (5568) | Calibrated dates on wheat  (2σ) | Reference |
| --- | --- | --- | --- | --- | --- | --- | --- | --- | --- | --- | --- | --- | --- | --- | --- |
|  |  | count | ubiquity | count | ubiquity | count | ubiquity | count | ubiquity | count | ubiquity |  |  |  |  |
| Xiazhai | Henan |  |  |  |  |  |  |  |  |  |  |  | Earliest: 2510±25 | Earliest: 789-542BC (see Table 1) | This study |
| Xinzhai | Henan | 5 | 0.90% |  |  |  |  |  |  |  |  |  |  | - | Zhao 2011; Zhong et al. 2016 |
| Wadian | Henan | 8 | 4.32% |  |  |  |  |  |  | 1 |  |  |  | Historical period | Liu and Fang 2010; Zhao 2015b |
| Xijincheng | Henan | 1 | 3.30% |  |  |  |  |  |  |  |  |  |  | - | Chen et al. 2010 |
| Baligang | Henan | 6 | 6.35% |  |  |  |  | 19 |  | 25 | 66.67% | BA081055 | 2500±35 | 790-420BC | Deng et al. 2015; This study |
|  |  |  |  |  |  |  |  |  |  |  |  | UGAMS#  27671 | 3670±25 | 2137-1966BC |  |
| Dalaidian | Henan | 2 | 2.27% |  |  |  |  |  |  |  |  |  |  |  | Wu 2016 |
| Pingliangtai | Henan | 1 | 5.90% |  |  |  |  |  |  |  |  |  |  |  | Deng and Qin 2017 |
| Matun* | Henan | 1 | - |  |  |  |  |  |  |  |  |  |  |  | Zhang et al. 2014 |
| Wangchenggang | Henan |  |  | 1 |  | 191(E^3^) | 100% (E) |  |  | 65 | 47.60% | QAS1302 | 3155±30 | 1501-1320 BC; | Liu et al. 2016; Zhao and Fang 2007 |
|  |  |  |  |  |  |  |  |  |  |  |  | QAS1303 | 3205±25 | 1519-1426 BC; |  |
|  |  |  |  |  |  |  |  |  |  |  |  | QAS1304 | 3210±40 | 1608-1412 BC; |  |
|  |  |  |  |  |  | 60(L^4^) |  |  |  |  |  | QAS1305 | 3180±28 | 1501-1415 BC; |  |
|  |  |  |  |  |  |  |  |  |  |  |  | QAS1306 | 2475±20 | 764-516 BC |  |
| Guchengzhai | Henan |  |  | 1 | 11.11% | 6（E） | 27.78%（E） |  |  |  |  |  |  | - | Chen et al. 2012 |
|  |  |  |  |  |  | 2（L） | 12.5%（L） |  |  |  |  |  |  |  |  |
| Erlitou | Henan |  |  | 3 |  | 5（E） |  |  |  |  |  |  |  | - | Zhao 2015a |
| Zaojiaoshu | Henan |  |  | 16+ |  |  |  |  |  |  |  |  |  | - | Archaeological excavation team of Luoyang city, 2002 |
| Fengzhai* | Henan |  |  |  |  | 92 |  |  |  |  |  |  |  | - | Lee et al. 2007 |
| Tianposhuiku* | Henan |  |  |  |  | 172 |  |  |  |  |  |  |  | - | Lee et al. 2007 |
| Yanshishangcheng | Henan |  |  |  |  | √^2^ |  |  |  |  |  | QAS1326 | 3140±20 | 1492-1319 BC | Liu et al. 2016 |
| Zhengzhoushangcheng | Henan |  |  |  |  | 91（E） | 52.17%（E） | |  |  |  |  |  |  | Jia 2011 |
| Nanwa | Henan |  |  | 6 | 3.66% | 16（L） | 32%（L） |  |  | 2 |  |  |  | - | Wu et al. 2014 |
| Dongzhao | Henan |  |  | 7 | 8.50% | 103（E） | 59%（E） |  |  |  |  | UGAMS#23483 | 3110±25 | 1434-1298BC | Yang et al. 2017 |
| Shaochai* | Henan |  |  | 8 |  |  |  |  |  |  |  |  |  |  | Zhang et al. 2014 |
| Jingyanggang* | Henan |  |  | 2 |  |  |  |  |  |  |  |  |  |  | Zhang et al. 2014 |
| Feiyaonan* | Henan |  |  |  |  | 7 |  |  |  |  |  | BA10574 | 2840±25 | 1120-910 BC; | Zhang et al. 2014 |
|  |  |  |  |  |  |  |  |  |  |  |  | BA10575 | 3000±30 | 1380-1120BC |  |
| Guanzhuang* | Henan |  |  |  |  |  |  | 109 | 34.29% | 2 |  |  |  |  | Lan and Chen 2014 |
| Gaoya* | Henan |  |  |  |  |  |  |  |  | 1 |  |  |  |  | Zhang et al. 2014 |
| Dugangsi* | Henan |  |  |  |  | 2(L) |  |  |  |  |  |  |  |  | Fuller and Zhang 2007 |
| Anban* | Shaanxi | 3 |  |  |  |  |  |  |  |  |  |  |  | - | Liu 2014 |
| Wangjiazui | Shaanxi | 1 |  |  |  | 120（L） |  |  |  |  |  | QAS1324 | 2895±20 | 1190-1007 BC | Liu et al. 2016; Zhao and Xu 2004 |
| Zhuangli | Shaanxi |  |  |  |  |  |  | 533 | 94.44% |  |  |  |  |  | Zhou yuan Archaeological Team 2011 |
| Nansha* | Shaanxi |  |  |  |  |  | √ |  |  |  |  | OZM459 | 3260±35 | 1618-1450 BC | Dodson et al. 2013 |
|  |  |  |  |  |  |  |  |  |  |  |  | OZM460 | 3275±30 | 1626-1461 BC |  |
|  |  |  |  |  |  |  |  |  |  |  |  | OZM458 | 3300±30 | 1663-1501 BC |  |
| Donggao* | Shaanxi |  |  |  |  |  |  |  | √ |  |  | OZM464 | 960±35 | 1017-1159 AD | Dodson et al. 2013 |
|  |  |  |  |  |  |  |  |  |  |  |  | OZM463 | 2850±35 | 1122-919 BC |  |
| Shangguancun* | Shaanxi |  |  |  |  | √ |  |  |  |  |  | OSL434 | 1320±39 | 649-773 AD | Dodson et al. 2013 |
| Shuinan 3* | Shanxi | 4 |  |  |  |  |  |  |  |  |  |  |  |  | Song et al 2017 |
| Hucun 4* | Shanxi | 1 |  |  |  |  |  |  |  |  |  |  |  |  | Song et al 2017 |
| Nan’gao* | Shanxi | 10 |  |  |  |  |  |  |  |  |  | UGAMS#27673 | 1580±20 | 421-539AD | Jiang 2017; this study |
| Nanjie* | Shanxi | 16 |  |  |  |  |  |  |  |  |  | UGAMS#27672 | 100±20 | 1691-1925AD | Jiang 2017;this study |
| Baishi* | Shanxi | 1 |  |  |  |  |  |  |  |  |  |  |  |  | Jiang 2017 |

**Notes**

1. all periods are based on associated material culture affiliations

2.√indicates presence reported without quantity details

3. E =Early Shang period;

4. L= Late Shang period.

**References cited in Table S2**

Archaeological Team of Luoyang City (2002) Archaeological excavation report of the Zaojiaoshu site in Luoyang, Science Press, Beijing.

Chen X, Wang L, Wang Q (2010) Flotation results from the excavation of Xijincheng site in 2006 and 2007. Huaxia Archaeology 3:67-76

Chen W, Zhang J, Cai Q (2012) Analysis of the plant remains from the Guchengzhai city site in Xinmi, Henan. Huaxia Archaeology:54-62

Deng Z, Qin L, Gao Y, Weisskopf AR, Zhang C, Fuller DQ (2015) From Early Domesticated Rice of the Middle Yangtze Basin to Millet, Rice and Wheat Agriculture: Archaeobotanical Macro-Remains from Baligang, Nanyang Basin, Central China (6700–500 BC). PloS one 10:e0139885

Deng Z, Qin L (2017) A comparative study on the agriculture structure of the central plains in the Longshan period. Huaxia Archaeology 3:98-108

Dodson JR, Li X, Zhou X, Zhao K, Sun N, Atahan P (2013) Origin and spread of wheat in China Quaternary Science Review. 72:108-111

Fuller DQ, Zhang H (2007) A preliminary report of the survey archaeobotany of the upper Ying Valley (Henan Province). In: School of archaeology and museology Peking University, Henan provincial institute of Archaeology (eds) Archaeological discovery and research at the Wangchenggang site in Dengfeng (2002–2005). Great Elephant Press, Zhengzhou, pp 916-958

Jia S (2011) Research of Charred Plant Remains from Ruins of Shang City in Zhengzhou. Master Dissertation, University of Science and Technology of China

Jiang Y (2017) The Agricultural Economy and Society of Northern China during the Longshan Period. PhD dissertation, Peking Univesity

Lan W, Chen Z (2014) Analysis of macro plant remains from the Guanzhuang site, Xingyang city. East Asia Archaeology 11:402-406

Lee G-A, Crawford GW, Liu L, Chen X (2007) Plants and people from the Early Neolithic to Shang periods in North China Proceedings of the National Academy of Sciences 104:1087-1092

Liu C, Fang Y (2010) Analysis of Plant remains from Wadian site, Henan province. Relics from South 4:55-64

Liu X (2014) Research on the plant remains from the 2010 excavation of AnBan Site, Shaanxi Province. Master Dissertation, Northwest Universty

Liu X et al. (2016) The virtues of small grain size: Potential pathways to a distinguishing feature of Asian wheats. Quaternary International 426:107-119

Song J, Wang L, Fuller DQ (2017). A regional case in the development of agriculture and crop processing in northern China from the Neolithic to Bronze Age: archaeobotanical evidence from the Sushui River survey, Shanxi province. Archaeological and Anthropological Sciences, doi:10.1007/s12520-017-0551-0

Wu W, Zhang J, Jin G (2014) Archaeobotanical evidence for the ancient agriculture from Erlitou to Han dynasty at the Nanwa site, Dengfeng, Henan. Cultural Relics in Central China 1:109-117

Wu X (2016) The research of plant remains in Longshan period from Dalaidian site in Henan. Master Dissertation, Shandong University

Yang Y et al. (2017) Characteristics and development of agriculture during Xia and Shang dynasties based on carbonized plant analysis at the Dongzhao Site, Central China. Acta Anthropologica Sinica 36:119-130

Zhang J, Xia Z, Zhang X (2014) Research on charred plant remains from the Neolithic to the Bronze Age in Luoyang Basin. Chinese Science Bulletin 59:3388-3397

Zhao Z (2011) Characteristics of agricultural economy during the formation of ancient Chinese civilization. Journal of National Museum of China 1:019-031

Zhao Z (2015a) Flotation results of the Erlitou site. In: Institute of Archaeology CAoSS (ed) Erlitou (1999-2006). Cultural Relics Press, Beijing, pp 1295-1313

Zhao Z (2015b) Research on the spread of wheat into China: arcaheobotanical evidences. Relics from South 3:44-52

Zhao Z, Fang Y (2007) Aalysis on the Flotation results from the Wangchenggang site, Dengfeng City. Huaxia Archaeology 2:78-89

Zhao Z, Xu L (2004) Analysis on the Flotation results of Zhouyuan (Wangjiazui locality), Shaanxi province. Cultural Relics 10:89-96

Zhong H, Zhao C, Wei J, Zhao Z (2016) Analysis of flotation results from the excavation of Xinzhai site in 2014. Agricultural Archaeology 1:21-29

Zhouyuan Archaeological Team (2011) The Excavation of the Bronze Casting Remains in Locus West of Zhuangli Village at Zhouyuan Site in the Springs of 2003 and 2004. Acta Archaeologica Sinica 2:245-300
